# Applicability of Western protocols in resource‐limited setting: Real‐world data of long‐term outcome of intensive treatment of adult acute myeloid leukaemia in Sri Lanka

**DOI:** 10.1002/jha2.191

**Published:** 2021-05-13

**Authors:** Saman Hewamana, Lakmali Kandabadage, Thurairajah Skandarajah, Natasha Pieris, Eranga Perera, Mahesh Harischandra, Ananda Wijewickrama, Chandana Wickramarathna, Gnani Somasundaram, Vadivelu Srinivasan, Surjit Somiah, Priyankara Jayawardena, Mehendra Perera, Dehan Gunasekera, Chathuri Jayasinghe, Godvin Constantine, Sanjeewa Munasinghe, Chandu De Silva, Bandula Wijesiriwardena, Jayantha Balawardena

**Affiliations:** ^1^ Clinical Haematology Unit Lanka Hospitals Colombo Sri Lanka; ^2^ National Cancer Institute Colombo Sri Lanka; ^3^ Asiri Surgical Hospital Colombo Sri Lanka; ^4^ Lanka Hospitals Colombo Sri Lanka; ^5^ National Institute of Infectious Disease Angoda Sri Lanka; ^6^ Faculty of Medicine University of Ruhuna Matara Sri Lanka; ^7^ National Hospital of Sri Lanka Colombo Sri Lanka; ^8^ University of Sri Jayewardenepura Colombo Sri Lanka; ^9^ Army Hospital Colombo Sri Lanka; ^10^ Faculty of Medicine University of Colombo Colombo Sri Lanka; ^11^ Sir John Kotelawala Defence University, Werahera Colombo Sri Lanka

**Keywords:** AML, developing countries, Sri Lanka, survival

## Abstract

There are no published data on long‐term survival and applicability of treatment protocols from developed countries in acute myeloid leukaemia (AML) in Sri Lanka. Eighty‐seven AML patients were reviewed; there were 56 newly diagnosed patients between 18 and 65 years. Thirty‐one out of 33 who started treatment achieved complete remission after first cycle of treatment. The induction mortality was one of 33. Twelve out of 20 patients who completed treatment are alive at the time of analysis. The estimated 5‐year overall survival rate is 0.629. Strict infection control and treatment and superior clinical experience may have contributed towards better outcome.

## 1 INTRODUCTION

Acute myeloid leukaemia (AML) is a haematological malignancy, which is almost always fatal without treatment with survival ranging from few days to a few weeks [1].

Sri Lanka is a developing country with diverse health care structure, without dedicated transplant facilities or access to novel antileukaemic agents at the time this study was started.

Sri Lanka lacks the necessary technology and expertise in performing allogeneic transplants and the cost of these in neighbouring Singapore and India are prohibitive. Crude incidence rate of ‘Leukaemia’ in Sri Lanka is 3.6 in males and 3.1 in females (source: Cancer Incidence Data Sri Lanka 2011; Publication by Government Cancer Control Programme).

We established Lanka Hospital Blood Cancer Centre (LHBCC) in a self‐financing hospital in Sri Lanka in collaboration with colleagues in government subsidised hospitals with designated space, staff and a strategy to treat blood cancers using treatment protocols from the United Kingdom (UK). In addition, this centre was used for training purposes of first haemato‐oncology trainees from government‐subsidised hospitals.

The aim of the study was to analyse patient and disease characteristics and evaluate survival parameters in patients diagnosed with AML treated in line with treatment and supportive care protocols from the UK.

## 2 MATERIALS AND METHODS

Approval was obtained from the Lanka Hospitals medical research and the ethics committee for the study. All patients with a diagnosis of AML who presented to LHBCC from 1 May 2013 to 1 April 2020 were included in the analysis to check the outcome of treatment/services delivered.

They received induction with cytarabine (Cytosar, Pfizer) and daunorubicin (Daunomycin, Pfizer) (DA), followed by two courses of high‐dose cytarabine 3 g/m^2^. Data were analysed using GraphPad Prism 8.0 software (GraphPad Software, San Diego, CA).

## 3 RESULTS

A total of 87 patients with a diagnosis of AML were reviewed; 56 newly diagnosed patients between 18 and 65 years (young adult AML) were offered intensive chemotherapy. However, only 33 patients opted to start treatment in our centre (age range 19–65; 17 females and 16 males). These patients had genetic analysis for t(15;17), t(8;21) (three positive) and inv(16), while 12 had above and *FLT3* internal tandem duplication (*ITD*) mutation (one positive and 11 negative) and *NPM1* mutation (one positive and 11 negative).

Thirty‐one out of 33 who opted to start treatment in LHBCC achieved CR after first cycle of treatment (31/33). There was one death during first cycle of induction chemotherapy (induction mortality one of 1/33) and one did not show desired response (blasts of less than 10% and considered as primary resistant AML). However, nine patients decided to stop treatment after induction cycle 1, and one patient decided to stop treatment after induction cycle 2. One of 21 patients undergoing consolidation therapy died during consolidation; consolidation mortality of 1/21. The complete response rate of the population that continued treatment in LHBCC was 20/23 (two deaths during treatment and one primary resistant AML). Twelve out of 20 patients who completed the intended treatment are alive (11 in CR1 and one in CR2) at the time of analysis (31 December 2020). There were six early relapses and one late relapse, while two patients died due to unrelated causes while in complete remission. At a median follow‐up of 47.99 months, median OS for the whole cohort is 11.83 months. However, subanalysis showed median OS of patients who decided to stop treatment is mere 1.68 months, while patients who completed treatment have not yet reached with restricted mean OS of 66.32 months (estimated 5‐year OS of 0.629). Two patients who died during the therapy are not included in the final survival analysis but discussed in the treatment‐related mortality.

**FIGURE 1:**
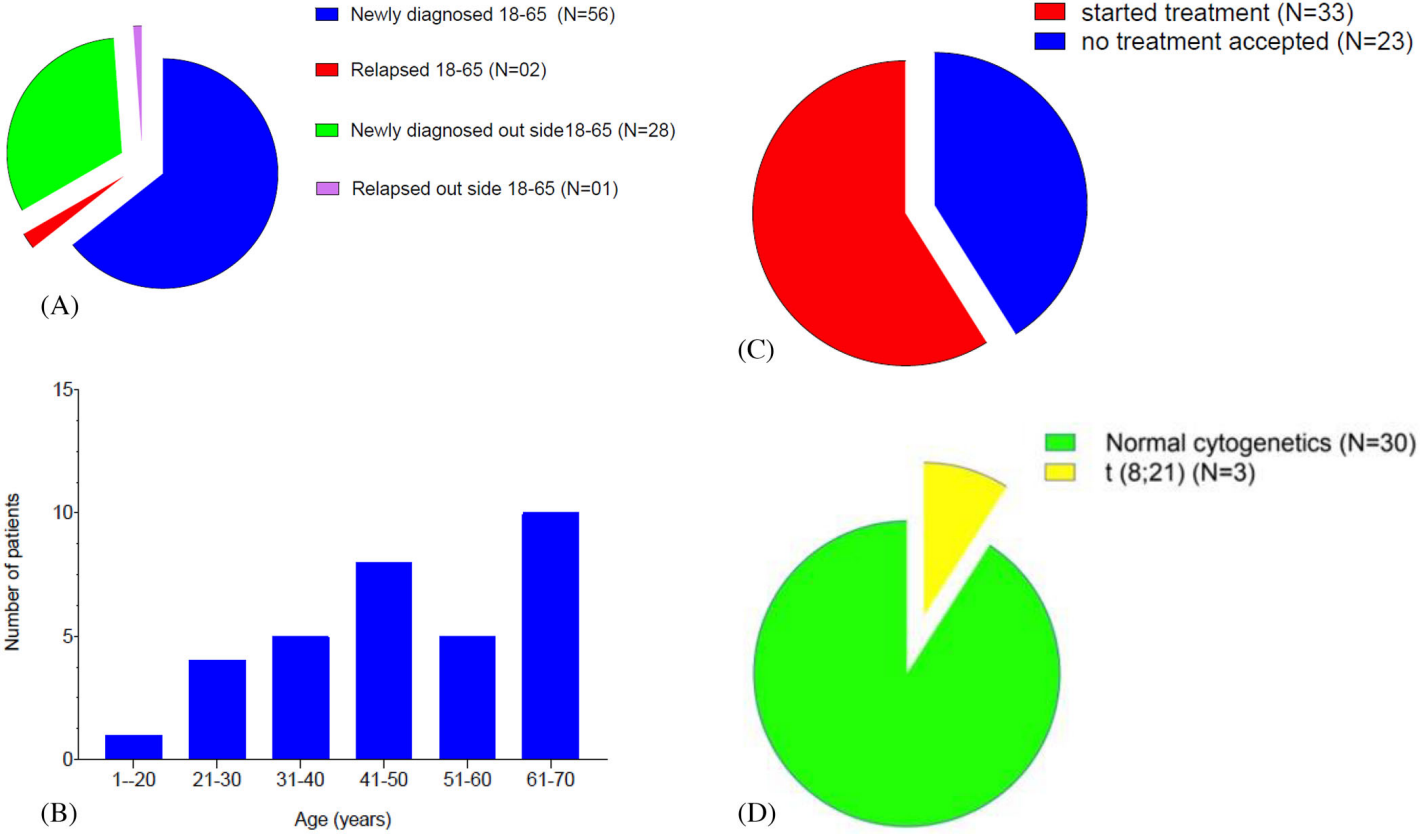
(A and B) Age distribution of newly diagnosed cases of acute myeloid leukaemia. (C) Proportion of newly diagnosed patients that opted for treatment. (D) Genetic analysis of patients who opted for treatment in LHBCC

**FIGURE 2:**
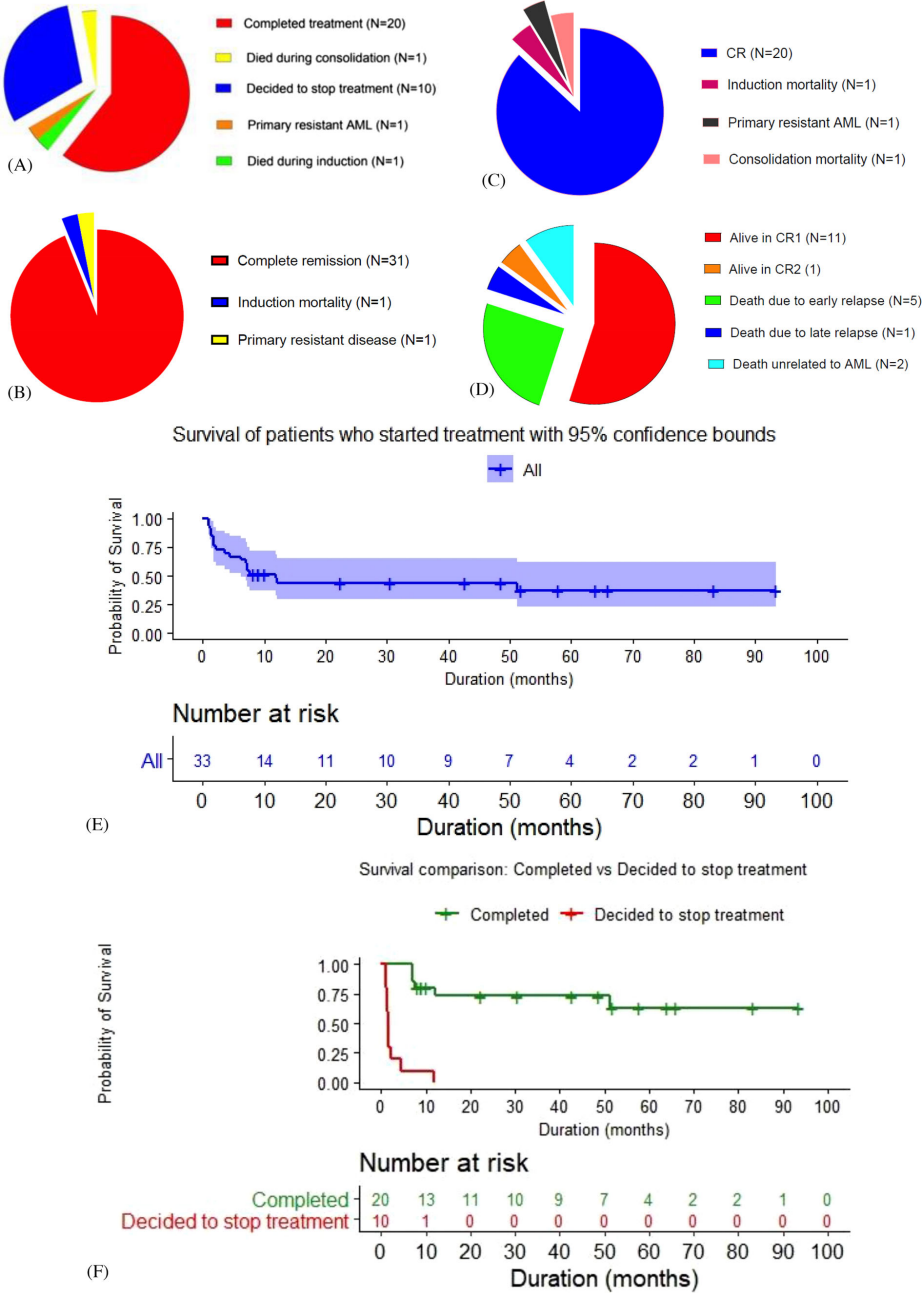
(A) Outcome of patients who started treatment in LHBCC. (B) Outcome after induction phase 1. (C) Outcome of patients who continued treatment. (D) Outcome of patients who achieved CR after four cycles of treatment. Survival of patients who started treatment in LHBCC (E) and comparative survival analysis of patients who continued treatment versus who stopped treatment (F)

Patient and disease characteristics are summarised in Figure 1 and Table 1; outcome data are given in Figure 2 and Table 2.

**TABLE 1:** Patient and disease characteristics of newly diagnosed young adult AML reviewed in LHBCC

**(A) Presentation and age group of AML reviewed in LHBCC (*N* = 87)**		
	*N*	%
Newly diagnosed 18–65 years	56	64.37
Relapsed 18–65 years	02	2.30
Newly diagnosed outside 18–65 years	28	32.18
Relapsed outside 18–65 years	1	1.15

**(B) Age distribution of young adult AML started treatment in LHBCC (*N* = 33)**		
Age group (years)	*N*	%
1–20	1	3.04
21–30	4	12.12
31–40	5	15.15
41–50	8	24.24
51–60	5	15.15
61–70	10	30.30

**(C) Proportion of newly diagnosed young adult AML started treatment in LHBCC (*N* = 56)**		
	*N*	%
Started treatment	33	58.93
No treatment accepted	23	41.07

**(D) Genetic analysis of young adult AML started treatment in LHBCC (*N* = 33)**		
Genetics t(8;21)/t(15;17)/inv(16)	*N*	%
t(8;21)	3	9.09
Normal cytogenetics	30	90.91

*Note*: (A and B) Age distribution of newly diagnosed cases of acute myeloid leukaemia. (C) Proportion of newly diagnosed patients that opted for treatment. (D) Genetic analysis of patients who opted for treatment in LHBCC.

**TABLE 2:** Outcome of newly diagnosed young adult AML treated in LHBCC

**(A) Outcome of young adult AML started treatment in LHBCC (*N* = 33)**		
Outcome	*N*	%
Completed treatment	20	60.61
Decided to stop treatment after induction	10	30.30
Primary resistant AML	1	3.03
Died during induction	1	3.03
Died during consolidation	1	3.03

**(B) Outcome of induction 1 for young adult AML in LHBCC (*N* = 33)**		
	*N*	%
Complete remission	31	93.94
Induction mortality	1	3.03
Primary resistant disease	1	3.03

**(C) Outcome of young adult AML continued treatment in LHBCC (*N* = 23)**		
	*N*	%
Complete remission	20	87.00
Induction mortality	1	4.37
Primary resistant disease	1	4.37
Consolidation mortality	1	4.37

**(D) Outcomes of young adult AML in postconsolidation CR in LHBCC (*N* = 20)**		
Outcome	*N*	%
Alive in CR1	11	55
Death due to early relapse	05	25
Death due to late relapse	1	5
Alive in CR2	1	5
Death unrelated to AML	2	10

**(E) Number at risk: young adult AML started treatment in LHBCC (*N* = 33)**	
Time (in months)	Number at risk
1.00	33
1.03	32
1.20	31
1.30	30
1.33	29
1.67	28
1.70	27
1.77	26
2.27	25
3.57	24
4.37	23
6.27	22
7.10	21
7.13	20
7.70	18
11.83	13
12.17	12
51.23	7

**(F1) Number at risk: young adult AML in postconsolidation CR in LHBCC (*N* = 20)**	
Time (in months)	Number at risk
7.10	20
7.13	19
7.70	17
12.17	12
51.23	7

**(F2) Number at risk: young adult AML decided to stop treatment in LHBCC (*N* = 10)**	
Time (in months)	Number at risk
1.03	10
1.20	9
1.30	8
1.33	7
1.67	6
1.70	5
1.77	4
2.27	3
4.37	2
11.83	1

*Note*: (A) Outcome of patients who started treatment in LHBCC. (B) Outcome after induction phase 1. (C) Outcome of patients who continued treatment. (D) Outcome of patients who achieved CR after four cycles of treatment. Number at risk of (E) started treatment (F1) achieved CR and (F2) decided to stop treatment in LHBCC.

## 4 DISCUSSION

AML is a deadly disease in the West and a deadly and a costly challenge in the developing countries [2]. Several studies have demonstrated the association between the socioeconomic status and the access to and the distribution of modalities of AML treatment [3]. Sri Lanka is a developing country with a diverse health care system. Unlike in the UK, different hospitals in Sri Lanka are likely to have different approaches to the same disease and also significant heterogeneity with regards to diagnostic and treatment facilities, access to trained personnel and supportive care.

### 4.1 Poor compliance with treatment due to lack of insight and financial reasons

It has been shown that survival and treatment options available depend on the insurance status and country of residence, and South Asian data showed poor compliance rates compared to Western trials [4, 5, 6]. Our data show that 33/56 (58%) initially agreed for treatment compared to 29% reported by Philip et al., but further nine decided to stop treatment after the first cycle. Total number of patients who did not proceed with the consolidation chemotherapy was 10 (10/31) due to financial reasons. In the developed countries, the cost of AML treatment is between US $80,000 and US $150,000 per patient and regional data showed it to be around US $32,500, which may be many times of one's annual income [4, 7, 8].

As reported by Burnet et al. in AML15 study, 101 patients given induction did not receive consolidation and had a poor survival compared to the ones who completed treatment (35% vs. 54%, *p* < .001) [15]. However, the reason for not continuing treatment in the West is due to delayed haematopoietic recovery compared to financial reasons in low‐income countries. People who decided to stop treatment in LHBCC had a median survival of mere 1.65 months, but the median survival for the ones who continued care has not reached at the time of the analysis.

### 4.2 Lower induction and consolidation mortality due to strict protocols and guidelines adoption

Guidelines make health care more consistent and efficient and reduce mortality and morbidity, but there are no well‐designed guidelines in developing countries [9, 10]. The induction death rate was 25%, 17% and 18.4% in the following regional publications by Philip et al., Kalaiyarasi et al. and Bahl et al. [4, 6, 11] in the same age group. In comparison, we lost one patient (3%) during induction and another during the consolidation (5%). In the AML15 trial, CR rate of DA was 84% [15] compared to 31/33 (93%) after course 1 of DA in our cohort. We have excluded patients outside 18–65 years range, making it difficult to do a proper comparison. Two patients died due to sepsis but in general our lower rate of treatment‐related mortality was due to strict infection prevention measures and aggressive treatment, which is one of the most important parameters in AML care as published before [12]. Furthermore, it has been shown that improved survival in AML may be due to advances in supportive care and superior clinical experience [13].

We used similar antifungal and antimicrobial prophylaxis as used in the UK AML trials, and neutropenic sepsis was treated using modified Western guidelines.

### 4.3 Better survival figures in patients who continued treatment

Even in the countries with best facilities, 5‐year OS rate in AML ranges between 25% and 40% for the group receiving intensive treatment [5, 14]. However, Burnet et al. reported in AML15, an 8‐year survival rate of 47% for patients who received two cycles of DA/ADE and two cycles of consolidation. The 5‐year survival from diagnosis in a group of patients treated with the same protocol and with an almost identical age distribution to our patients in the AML15 study was above 40%. We used only high‐dose cytarabine consolidation as Amsacrine for other options are not available in Sri Lanka and also we believe patients are in the low‐ and intermediate‐risk groups on the limited genetic analysis available. In addition, other consolidation options were shown to need more supportive care [15]. Subanalysis of patients who continued treatment in our cohort showed 5‐year estimated OS rate of 62.9%. Comparative regional data has shown OS of 20.6 months and estimated 5‐year OS rate of 35.5% [11]. Our treatment success is likely due to uniform treatment protocols, having full‐time in‐house clinical haematologist/haemato‐oncologists, following Western protocols to treat AML and treatment‐related complications and comprehensive supportive care. We acknowledge that a limitation of this study is the very small sample size, which could have resulted in some selection bias towards better outcome.

## 5 CONCLUSION

This is the only documented study related to outcome and successful applicability of Western treatment and supportive care protocols to Sri Lankan patients with AML. Though this can be considered as a small pilot study, we believe this published data will help to benchmark and in the development of the speciality of blood cancer care in the local setting.

## CONFLICT OF INTEREST

The authors declare that there is no conflict of interest.

## AUTHOR CONTRIBUTIONS

Performed research, data analysis, wrote paper, and provided health care to patients: Saman Hewamana. Performed research, data analysis and provided health care to patients: Lakmali Kandabadage. Performed research and provided health care to patients: Thurairajah Skandarajah, Natasha Pieris, Eranga Perera, Mahesh Harischandra, Ananda Wijewickrama, Chandana Wickramarathna, Gnani Somasundaram, Vadivelu Srinivasan, Sujith Somaigh, Priyankara Jayawardena, Mehendra Perera, Dehan Gunasekera, Godvin Constantine, Sanjeewa Munasinghe and Bandula Wijesiriwardana. Data analysis: Chathuri Jayasinghe. Performed diagnostic workup, designed the study and reviewed manuscript: Chandu De Silva. Performed research, reviewed manuscript and provided health care to patients: Jayantha Balawardena. All the authors reviewed the manuscript and agreed on its contents.

## Supporting information

Supporting Information

Supporting Information

## Data Availability

The data that support the findings of this study are available from the corresponding author upon reasonable request.
