# Supplementary figures and images for: Applicability of Western protocols in resource‐limited setting: Real‐world data of long‐term outcome of intensive treatment of adult acute myeloid leukaemia in Sri Lanka
Source: EJHaem. 2021 May 13;2(3):555–61. doi: 10.1002/jha2.191 (PMC9176152; doi:10.1002/jha2.191)

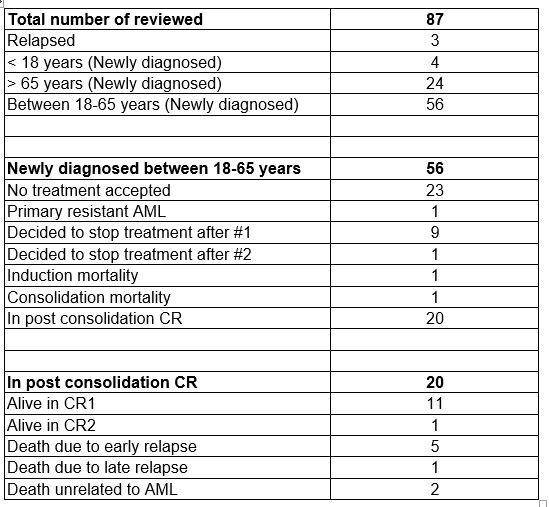

Supplement: Supplementary file 1 — Supporting Information [file JHA2-2-555-s002.JPG]

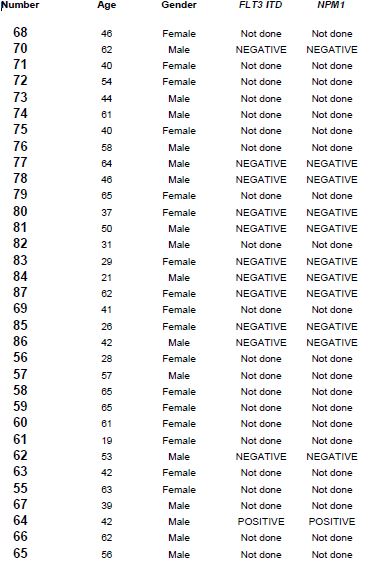

Supplement: Supplementary file 2 — Supporting Information [file JHA2-2-555-s001.JPG]
